# Aberrations in the Iron Regulatory Gene Signature Are Associated with Decreased Survival in Diffuse Infiltrating Gliomas

**DOI:** 10.1371/journal.pone.0166593

**Published:** 2016-11-29

**Authors:** Cody Weston, Joe Klobusicky, Jennifer Weston, James Connor, Steven A. Toms, Nicholas F. Marko

**Affiliations:** 1 College of Medicine. Penn State Milton S. Hershey Medical Center, Hershey, Pennsylvania, United States of America; 2 Department of Data Science. Geisinger Medical Center, Danville, Pennsylvania, United States of America; 3 Department of Public Health Sciences. Penn State Milton S. Hershey Medical Center, Hershey, Pennsylvania, United States of America; 4 Department of Neurosurgery. Geisinger Medical Center, Danville, Pennsylvania, United States of America; 5 Department of Surgery. Pennsylvania State School of Medicine, Danville, Pennsylvania, United States of America; University of Pécs Medical School, HUNGARY

## Abstract

Iron is a tightly regulated micronutrient with no physiologic means of elimination and is necessary for cell division in normal tissue. Recent evidence suggests that dysregulation of iron regulatory proteins may play a role in cancer pathophysiology. We use public data from The Cancer Genome Atlas (TCGA) to study the association between survival and expression levels of 61 genes coding for iron regulatory proteins in patients with World Health Organization Grade II-III gliomas. Using a feature selection algorithm we identified a novel, optimized subset of eight iron regulatory genes (STEAP3, HFE, TMPRSS6, SFXN1, TFRC, UROS, SLC11A2, and STEAP4) whose differential expression defines two phenotypic groups with median survival differences of 52.3 months for patients with grade II gliomas (25.9 vs. 78.2 months, p< 10^−3^), 43.5 months for patients with grade III gliomas (43.9 vs. 87.4 months, p = 0.025), and 54.0 months when considering both grade II and III gliomas (79.9 vs. 25.9 months, p < 10^−5^).

## Introduction

Gliomas are the most common intrinsic brain tumors, with an incidence of 21.42 per 100,000 individuals and an overall mortality rate of 4.26 per 100,000 individuals [1]. World Health Organization (WHO) grade II/III gliomas, sometimes termed “lower-grade gliomas” (LGG) [2,3], eventually progress to glioblastomas (WHO grade IV) which have a median survival time of approximately 15 months with standard therapy [4]. Given the infiltrative nature, gradual progression, and inability of surgery or radiation to provide a cure to these tumors, there exist opportunities for targeted therapies aimed at slowing disease progression and prolonging survival. Finding putative molecular targets is an active area of research.

The potential association between dysregulated iron metabolism and cancer progression has recently come to prominence in translational oncology research. Dividing cells require iron for various enzymatic functions, notably ribonucleotide reductase, integral to the synthesis of deoxyribonucleotides [5]. On the other hand, free iron is capable of creating reactive oxygen species through the Fenton reaction [6,7]. Consequently, safe transport and storage of iron is necessary to limit oxidative stress to cells.

Recent studies have shown that when physiologic iron metabolism is disrupted, oxidative stress can drive mutagenesis and accelerate tumor progression [8,9]. Accordingly, therapeutic strategies related to iron metabolism, such as iron chelation therapy, are being tested in an attempt to improve prognosis in a variety of malignancies. Patients with iron metabolism disruptions may derive greater individual benefit from this approach [10]. Iron chelation therapy is not without adverse effects, including serious events such as reversible agranulocytosis and neutropenia [11]. Therefore, it would be clinically useful to identify patients prospectively who may benefit from iron chelation or other iron-related therapies.

A recently identified 16-gene iron regulatory gene signature (IRGS) has been shown to correlate with survival in patients with breast cancer [12]. A detailed description of the genes in the IRGS may be found in the S1 Text *Description of IRGS genes*. Recent translational neuro-oncology research has suggested that iron metabolism may also modulate tumor progression in CNS gliomas [13]. Variant forms of the gene encoding *HFE*, a key iron uptake mediator, are present with increased frequency in high-grade gliomas (glioblastoma, WHO grade IV) [14]. These findings appear to be clinically-significant, as one *HFE* variant appears to confer resistance to standard adjuvant glioma therapy (temozolomide + radiation) [15]. Similarly, polymorphisms in *HFE* have been shown to correlate with decreased survival in several forms of brain tumors, including both glioblastoma and metastatic brain tumors [16]. Based on these findings, we hypothesized that iron metabolism pathways may also be dysregulated in diffuse infiltrating gliomas and that differential expression of these genes may correlate with survival differences in LGG patients.

## Methods

### Datasets

Gene expression data for the complete set of 275 LGGs (as of Dec 1, 2015) was downloaded from The Cancer Genome Atlas (TCGA) using cBioPortal [17, 18] and the **cgdsr** package written for R [19]. The dataset used in this study is publicly available, and may be retrieved online through cbioportal.org. All data used is completely anonymized, and patients provided consent for the analysis of their tissue samples. This study has been approved through Geisinger Medical Center's institutional review board. Because of the longer survival of LGG patients, 218 (79.3%) of TCGA LGG patients were alive at the time of this analysis. Censored patients are defined as those alive at the end of the study, meaning that their true survival time extends beyond the study’s scope. Actual survival time was used for the remaining 57 (20.7%) patients.

Patients were further classified based on their specific grade. The dataset consists of 140 patients with grade III gliomas, of which 110 survived to the end of study, and 135 patients with grade II gliomas, of which 108 survived to the end of study. The vast majority of patients underwent some resection (267, or 97.1%). Of these 169 (61.5%) had a gross total resection, and 98 (35.6%) had a subtotal resection. Six patients (2.2%) underwent a biopsy, and grade data was not available for the remaining two patients (0.7%). In addition, 135 cases of glioblastoma multiforme (GBM) were similarly downloaded and examined as a comparator group. In contrast to lower grade glioma, the majority of patients (104, or 77.0%) were deceased by the end of study.

## Experimental Design

Retrospectively, 349 cases of LGG and 148 cases of GBM were evaluated for aberrant expression in 61 iron genes associated with iron metabolism. These 61 genes were selected based on the results of Miller and colleagues [12], who collected genes from iron metabolism categories in gene ontologies and literature review in a study of breast cancer.

For the purposes of our investigation, “aberrant expression” is defined an mRNA level with the absolute value of its z-score greater than 2, relative to the average expression level of the gene across all samples. Our analysis focuses specifically on identifying aberrant expression in these 61 iron metabolism genes that are associated with changes in overall survival.

### Survival Predictors

Several predictive methods can be used for estimating the length of patient survival. Following Kim and Bredel [20], we used a 1-nearest neighbor (1NN) algorithm and a Coxnet model, both of which are compared against a random selection baseline predictor.

#### Random Selection

As a baseline estimator, we used a leave-one-out random patient selection for survival times. Specifically, for each patient *P*, we sampled *T* uniformly from the set of survival times of all patients not including *P*, and defined our prediction as *T*. For assessing the average predictive errors of random selection, we performed random selection 100 times, and took the mean of estimates.

#### Coxnet model

As the number of genes open to analysis (approximately 10,000) greatly outnumbered our samples, we performed a feature selection algorithm of L1 and L2 penalties (elastic net regularization) applicable to several regression models developed by Simon et al. [21]. This algorithm uses a coordinate descent method for determining relative risks that maximize a likelihood with added regularization penalty. This penalty induces feature selection by assigning coefficient weights of zero to certain covariates. Simon and colleagues created the R package **Coxnet,** which applies this algorithm to determining relative risks in a regularized Cox regression model [21]. As shown by Kim and Bredel [20], Coxnet can be applied for feature selection when considering spaces of several thousand genes. However, as coordinate descent ultimately finds only local minima, it is not surprising that predictive power increases when restricted to sets of genes known to be correlated with cancer mortality. We selected 61 genes involved in iron regulation, which overlaps and expands upon the initial gene set considered by Miller and colleagues [12] in defining the IRGS.

#### Nearest Neighbor Predictor

For a particular sample *S*, the 1NN algorithm finds the sample *T* whose gene expressions are closest to *S* in the standard Euclidean distance. The prediction is then the survival of *T*. This method was used on the entire space of 61 iron regulatory genes, and also on the restricted space of 8 genes found through Coxnet feature selection. The 1NN algorithm has the advantage of not overfitting, instead placing equal importance on each gene in the Euclidean distance metric.

### Performance Metrics

Performance for random selection and 1NN is measured using the median of residuals, given by

$$|{\hat{Y}=Y}_{p}(i)-Y_{o}(i)|,$$

where *Y*_*p*_(*i*) and *Y*_*o*_(*i*) denote the *i*^*th*^ predicted and observed samples, and *N* denotes sample size. Measurements are given in terms of months of survival from diagnosis to date of death. For comparisons of residuals, we employ a nonparametric Mann-Whitney U test for the equivalence of medians, which has no assumptions of normality of distributions.

To compare predictive power of the Coxnet and 1NN algorithms, we used the Pearson correlation coefficient. For 1NN, we calculated the correlation coefficient *r*_1_ between observed survival times and predicted survival times. For the Coxnet model, we used the coefficient *r*_2_ as the correlation between patients’ relative risks and survival times. Survival analysis was conducted using the log-rank test for difference of survival curves.

For a given set of genes, we employ Kaplan-Meier curves for comparison of survival rates under a binary analysis. In our case, we separated patients with no gene aberrations and those with at least one aberrant gene. We defined an aberrant gene as a sample with a z-score with absolute value > 2. To test differences in the distribution of survival length, we employed the commonly used log-rank test. One advantage of the log-rank test is that it can incorporate censored data in its test statistic.

## Results

### Iron regulatory genes and model performance

We compared the two algorithms on the full space of 61 iron regulatory genes. The median score of residuals for random selection was 31.24. The 1NN performed significantly better, with a median residual of 17.97. The Mann-Whitney U test on equivalent of medians provides moderate evidence that residuals for 1NN are better than the baseline random model (p = 0.0502). That is, the 1NN method produces estimates of survival time with a median error less than that of random selection when analyzing the two groups using the 61 iron regulatory genes.

### Feature Selection: 1NN vs. Coxnet models

We now compare the predictive power of the Coxnet and 1NN methods. For the 1NN model, the correlation coefficient *r*_1_ using the full set of iron genes was 0.23. This produces higher correlation values than those found in Kim and Bredel [20], who found a correlation of 0.18 between observed and predicted survival of GBM survival times when employing 1NN over a collection of 12,042 genes. For the Coxnet algorithm, we performed 10-fold cross validation to determine *L*_1_ regularization parameters. Under an elastic net penalization, Coxnet reduced the number of parameters to eight genes. The correlation coefficient *r*_2_ for the Coxnet model was -0.30 (as we are comparing risk with survival times, we should expect a negative correlation). While this correlation was slightly weaker than the 1NN model, the removal of 53 of 61 genes produced stronger correlations when comparing with Kim and Bredel [20], who found a correlation of -0.22 using Coxnet applied to a collection of 12,042 genes, and a correlation of -0.24 when restricting to genes associated with cancer pathways. The Coxnet algorithm minimizes a penalized likelihood, and can assign multiple regression coefficients to exactly zero. By applying feature selection through choosing variables with nonzero coefficients, we reduced the 16 gene IRGS set to 4 genes and the 61 gene set to 8 genes (see Table 1).

**Table 1:** Regression coefficients for Coxnet.

Gene	Coefficient from 61-gene set for LGG	Coefficient from 16-gene set for LGG	Coefficient from 61-gene set for GBM
HFE	0.285	0.433	0.284
STEAP3	0.220	0	0.256
UROS	-0.187	0	0.135
SFXN1	-0.168	-0.178	-0.129
STEAP4	0.028	0	0.070
TMPRSS6	0.025	0	0.070
TFRC	.024	-.077	0.059
SLC11A2	-0.018	0	0
SLC25A37	0	0.009	0

Regression coefficients of genes chosen through Coxnet feature selection of the 61-gene set for LGG, 16-gene set for LGG, and 61 gene set for GBM. Genes are ordered by the absolute value of their regression coefficient for the 61 LGG gene set, where magnitudes provide a scale of predictive importance. A score of zero denotes that a gene is not selected for a corresponding gene set.

When employing 1NN model using only the 8 genes from feature selection, the correlation dropped to 0.14. The reduction in correlation can be explained by the variety of parameters provided in the proportional hazards model corresponding to each gene. In other words, under the Coxnet model, genes with higher predictive power are given more importance for determining survival times. This contrasts with 1NN, which, without any training phase, assumes all genes are equally related to survival lengths. Thus, the Coxnet model provided a method of reducing the number of genes with minor reductions in accuracy. Moreover, the Coxnet model is superior to the 1NN, as applying the same reduction produced undesirable results in the latter model.

### Kaplan-Meier Curve Analysis

**Fig 1:**
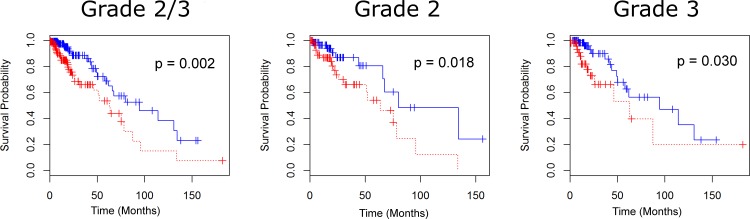
Survival for LGG patients under the IRGS (16-gene set). Survival curves for LGG patients with and without aberrations for grades 2 and 3 (A), grade 2 (B), and grade 3 (C). Under all grade considerations, the 16-gene set successfully splits the patient population, suggesting that a further analysis of gene selection may produce more significant differences in median survival times.

We now investigate how reducing gene sets through Coxnet can produce more powerful survival differences from the perspective of Kaplan-Meier curves. A summary of survival times, frequencies of populations with and without gene aberrations, and statistical significance is given in Table 2. First, we focus on the 16-gene IRGS identified by Miller et al. [12]. In 44.0% of the LGG cases (121 of the 275 samples) from the TCGA dataset there was at least one abnormally-expressed IRGS gene (denoted as “aberrant”). A Kaplan-Meier estimate comparing tumors with and without aberrations among the 16 genes of the IRGS showed significantly shorter overall survival (Fig 1A, Log-rank p = 0.002), and moderate evidence of difference in disease-free months (Log-rank p = 0.102). The median survival difference between cases without aberrations and cases with aberrations is 31.5 months, and a difference of 16.8 disease free months (note that only 42 uncensored samples were available for disease free survival, as 15 samples were missing data in this field). Survival times were also compared separately on grade II and grade III gliomas. For grade II gliomas, survival differences were 16.43 months (p = 0.054), and for grade III, survival differences were 31.54 months (p = 0.030).

**Table 2:** Summary statistics for overall survival under various glioma grades and gene selections.

	Median overall survival months: with aberration	Median overall survival months: with no aberration	Total number of patients with aberration	Total number of patients without aberration	p value
LGG/61 genes Grade 2 and 3	65.70	94.45	46	11	0.035
LGG/61 genes Grade 2	67.41	79.93	25	5	0.265
LGG/61 genes Grade 3	48.98	94.45	21	6	0.056
LGG/16 genes Grade 2 and 3	62.91	94.45	33	24	0.002
LGG/16 genes Grade 2	63.50	79.93	20	10	0.054
LGG/16 genes Grade 3	62.91	94.45	13	14	0.030
LGG/8 genes Grades 2 and3	25.89	79.93	23	34	<10^−5^
LGG/8 genes Grade 2	25.89	78.15	14	16	<10^−3^
LGG/8 genesGrade 3	43.86	87.39	9	18	0.025
GBM/61 genes	13.3	14.03	95	9	0.991
GBM/16 genes	12.94	14.03	62	42	0.430
GBM/8 genes	16.59	12.55	34	70	0.011

Median overall survival times, total numbers, and p values from the 61, 16, and 8 gene selections for both LGG (with further differentiation by grade) and GBM. While only weak evidence exists for differences of median overall survival times under the 61 gene set for Grade 2 patients, both the 16 gene set and 8 gene set produce statistically strong evidence for distinct subpopulations.

**Fig 2:**
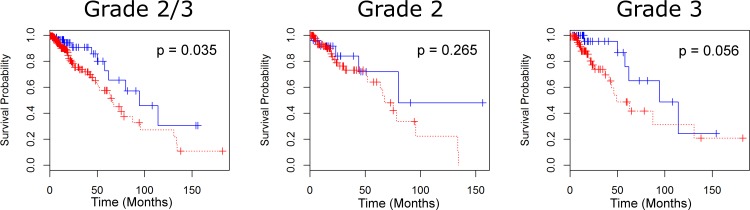
Survival for LGG patients under the full iron regulatory (61-gene) set. Survival curves for LGG patients with and without aberrations for grades 2 and 3 (A), grade 2 (B), and grade 3 (C). Moderate evidence for differentiating patient survival based on the 61 gene set exists for grade 2 and 3 gliomas, and grade 3. There is, however, weak evidence for the set successfully differentiating grade 2 gliomas. A finer subset analysis of the 61 gene set (Figs 1 and 4) reveals stronger markers for median survival times.

**Fig 3:**
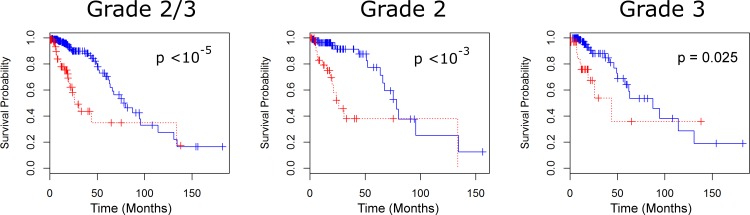
Survival for LGG patients under the feature selected (8-gene) set. Survival curves for LGG patients with and without aberrations for grades 2 and 3 (A), grade 2 (B), and grade 3 (C). In all cases, we observe that the 8-gene set discerns greater median survival times of populations than both the 16 and 61 gene sets.

For the full set of 61 iron regulating genes, 74.9% of samples have at least one aberrant gene. The separation criterion of having at least one aberrant gene of 61 gives less confidence than the sixteen gene set (p = 0.035), but similar differences in survival times (28.75 months) (see Fig 2). Restricting to specific grades, we observe only a 12.52 month difference in survival times with grade II gliomas and a low level of confidence (p = 0.265), but a much larger difference in times among grade III gliomas (45.47 months) with a higher level of confidence (p = 0.056). The greatest differences of survival times were obtained through limiting the analysis to our feature selected set of eight genes. With p < 10^−5^, we observe a median survival time difference of 54.03 months (see Fig 3A). Similar results were obtained for grade II gliomas (52.26 month difference with p < 10^−3^), and grade III gliomas (43.53 month difference with p = 0.025).

Next, we sought to determine the relative importance of these eight genes in LGG survival times through a greedy selection process, or one which optimizes survival times at each step. Among our eight identified genes, we selected the gene with the largest difference in survival times, namely *HFE*. The presence or absence of *HFE* predicted a difference in survival from LGG of 67.80 months. We then observed which of the other seven genes produced the largest difference in survival based on whether an aberration occurred in either a selected gene or *HFE*. This process continued recursively. Thus, given a set of *k* optimal genes, we selected a remaining gene that maximized survival time differences of *k* + 1 gene sets containing the optimal *k* gene set, based on whether any of the *k* + 1 genes is aberrant. Subsequent iterations from *HFE* produced, in general, lower survival results ending a median survival difference of 54.03 months after all eight genes were selected.

**Fig 4:**
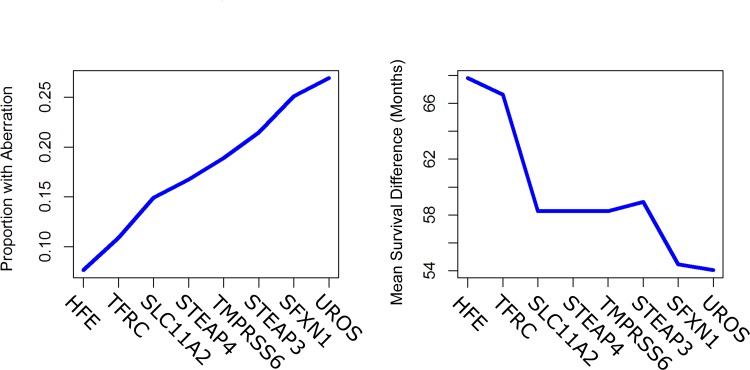
Greedy selection and survival times. Median survival (A) and aberrant gene frequency (B) of greedy gene selection process. The genes displayed the x-axis are aggregative, meaning that genes to the left are also included when considering survival differences. For example, the denotation “*TFRC”* corresponds to separation of populations for both *TFRC* and *HFE*. In (A), median survivals times begin to decrease after considering more than three genes. However, while greater differences in median survival exist when considering fewer genes, (B) exhibits that considering smaller gene sets also results in smaller percentages of patients with aberrant genes.

While smaller gene sets produced larger differences in survival, Fig 4B shows that percentages of patients with such aberrations grow considerably when selecting more genes, beginning with 7.6% of uncensored patients with an aberration of *HFE*, and ending with 26.9% of patients with at least one aberration in the eight gene set. For the log-rank test of equivalence of survival curves, each of the eight comparisons satisfied p < 10^−5^. To make a statement on the familywise error rate, or the probability of making at least one false error in multiple hypothesis tests, we used the Bonferroni correction factor: for *N* hypothesis tests with a p-value of *p*, we rejected the null hypothesis that at least one comparison was equivalent if we could reject each individual test with a p-value of p/N. While our end result from greedy selection produced 8-gene sets, the greedy selection procedure actually compares 45 tests (eight for selecting the first gene, seven for the second, and so forth). With this correction, survival comparisons pass the log-rank test with a p value of p = 45 × 10^−5^ < 0.001. Thus, with 99.9% confidence, all of the median survival differences produced in the greedy selection procedure are significantly different from 0.

### Feature Selection Applied to GBM Data

**Fig 5:**
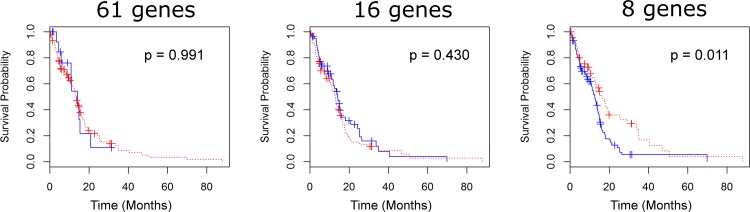
Survival for GBM patients. Survival curves of GBM patients with and without aberrations in feature selected 8-gene set (A), 16 gene IRGS (B), and full 61 gene set (C). In all cases, we fail to reject the null hypothesis of survival curve equivalence, suggesting that the of role iron regulation in GBM is different from that of LGG.

We applied similar statistical tests on data from 135 patients with GBM using TCGA database. While the GBM dataset contained fewer cases than the LGG dataset, the data is also mostly uncensored, with only 31 patients still alive at the conclusion of the study. Survival curves for GBM contrast sharply with LGG survival curves, as log rank tests for the 61 and 16 gene sets failed to produce statistically significant differences in survival times, with differences of 0.73 months for 61 genes (p = 0.991) and 1.09 months for 16 genes (p = 0.430) (see Fig 5). Interestingly, for 8 genes, there is statistical evidence that the presence of aberrations may produce *longer* survival times, with a difference of 16.59 months for those with aberrations, and 12.55 months for those without (p = 0.011).

Finally, a similar survival analysis was also applied to differentiate relevant genes of primary and secondary glioblastomas. Following Ohgaki and Kleihues [22], who found mutations of *IDH1* as a “decisive genetic signpost” between the two glioma types, samples were separated based on the gene’s mutation. As secondary GBMs are relatively rare, (~10% of GBMs per the literature [22] and 5.5% [6 of 240 tumors] with *IDH1* mutations in the TCGA data), we were unable to make any meaningful regression models with this heavily restricted subgroup. We also performed the same analyses on LGGs conditioned on patients with *IDH1* mutations, producing similar results. Specifically, for patients with *IDH1* mutations, we observed an average lifespan of 42.2 months for those with aberrations in the restricted eight-gene set, and 56.7 months without such aberrations. Patients with no *IDH1* mutations had a median lifetime of 18.6 months for those with aberrations in the 8-gene set, and 32.5 without such aberrations. However, the size of uncensored patients with *IDH1* aberrations (12 gliomas, or 4.4%) prevented us from making statistically significant statements about the difference of median survival time with this additional genetic criterion.

## Discussion

### Iron Gene Expression Predicts Survival

The present study shows that aberration in a set of eight iron-regulating genes is associated with decreased survival in patients with LGG. Given their capacity to predict substantial differences in patient survival, the eight genes identified in this analysis may have direct clinical utility. The modest number of genes makes the prospect of testing patients for aberrations feasible in a clinical setting. This could be of use in determining LGG prognosis and gives potential future implications for targeted therapy. The major caveat to these assertions is that the present study is retrospective and observational, which limits the applicability of these results at the current time. It is important to note that this study is mainly exploratory in nature, and thus will require confirmatory studies to verify the relation between iron genetics and glioma evolution. Prospective studies, for instance, would to determine whether these genes are influenced by treatment effects, apply to tumor subtypes, overlap with known prognostic factors, or simply correlate with enhanced sensitivity to disruptions in iron metabolism.

Additionally, a further study may provide information on the pronounced increase of survival difference between eight and sixteen gene subsets when considering patients with grade II versus grade III glioma. Specifically, for patients with grade II glioma, there is a 35.7 month increase in median survival difference for the eight gene subset compared to the sixteen gene subset. However, for patients with grade III glioma, the associated difference is only 12.0 months. The difference may possibly be explained by the more aggressive nature of grade III gliomas, but currently there is no such causal explanation.

### Biological Importance of the Eight Gene Subset

The largest factor in our model, *STEAP3*, also known as *TSAP6*, is a metalloreductase, converting iron and copper from trivalent to divalent cationic form [23]. In mouse models, STEAP3 overexpression in Raji cells, a line derived from Burkitt lymphoma, allowed the tumor growth to persist when low iron levels would normally encourage apoptosis [24]. Therefore, this gene may be responsible for a resilience to hypoferric conditions in multiple cancers, leading to a worse prognosis. Furthermore, *STEAP3* is associated with impaired exosome secretion, a process which has been implicated in antitumor immunity [25]. An understanding of the mechanism by which *STEAP3* function affects survival in LGG patients will require further study, though some combination of iron and immune modulatory effects are likely to contribute.

Although it is a smaller contributor to our model, STEAP4, also known as STAMP2, also affects survival. Like STEAP3, it is a metalloreductase acting on iron and copper. It has been shown to increase oxidative stress, and its disruption by siRNA knockdown significantly induced apoptosis in prostate cancer, and inhibited proliferation, colony formation, and growth. This was shown in cell culture and xenograft models using nanoliposomal delivery [26]. The entire STEAP family is already receiving some attention as a drug target, and our results may serve to reinforce its importance in human gliomas [27].

*TMPRSS6* is a membrane-bound regulator of iron homeostasis, implicated in the inhibition of hepcidin activation by hemojuvelin cleavage at the membrane [28]. Knockouts suffer from iron deficiency anemia, so it appears that its function is necessary for homeostatic levels of iron storage [29]. Others have found that variant forms of *TMPRSS6* were associated with increased risk of childhood ALL in males, so it may be common to multiple cancer types [30]. Similarly, *TFR2* is thought to be part of the iron sensing complex that regulates hepcidin [31]. When mutated, it disrupts normal neuronal function in the brain, which could affect the response of normal tissue to neoplastic insult [32]. *TFR2* is present in high levels in hepatocellular carcinoma, but there is little evidence for its presence in most cancers [33].

The emergence of *HFE* and *TFRC* as members of this predictive model is especially useful. First, the proteins derived from these two genes are known to interact, and disruption in function of either affects survival of patients with breast cancer [12,34]. Second, the highly polymorphic nature of *HFE* means that disruptions in normal *HFE* function are extremely common, and these findings support their clinical relevance in at least one cancer type. Therefore, screening could be of benefit to a relatively large subset of patients. In the US population, the two most common polymorphisms in *HFE*, *H63D* and *C282Y*, are seen in approximately 15% and 7% of the population, respectively [35]. Interestingly, recent work has also shown that the expression of TfR is a poor prognostic indicator in GBM, and that dysregulated iron metabolism including increased TfR expression and ferritin expression is associated with cancer stem cells in GBM [36] This suggests that the gene signature identified here is, at least in part, sorting cancers with more robust stem cell populations from cancers with fewer cancer stem cells.

SLC11A2 codes for DMT1, a transporter for divalent metals involved in iron absorption and another key molecule capable of modulating iron overload. For example, it’s overexpressed in the breast cancer line MCF-7 compared to the normal mammary cell line MCF-12A [37]. In human clinical populations, SLC11A2 has been associated with childhood ALL risk in males (OR = 1.52–2.60), and is being examined alongside TFRC and HFE for its role in modulating body iron load [38].

The UROS gene encodes for Uroporphyrinogen III synthase, part of the heme biosynthetic pathway. Until now, there has been little evidence of its relationship to nervous system cancers. However, increased activity has been shown to be associated with lymphoproliferative diseases in family studies [39]. Future studies are needed to better understand its role in gliomas and other cancers.

The presence of *SFXN1* in the model was more surprising. It remains poorly understood, but available evidence points to roles in mitochondrial function and iron transport [40, 41]. Its appearance in the survival model warrants a more pointed investigation into mitochondrial iron metabolism specifically.

### Selection of Predictive Methods

By pairing survival analysis with a penalized regression model, we have shown that a restricted set of genes has greater predictive power in LGG than the entire IRGS. While the full IRGS produces optimal predictive power in basic prediction algorithms, such as the 1NN model, the same gene set fails when taken as a binary analysis comparing survival curves of patients with and without at least one aberrant gene. Thus, in order to provide significant differences between survival curves, we must reduce the size of the gene space, which we have provided through penalized regression via Coxnet.

We have taken several measures to guard against overfitting of data. Our main tool is the Coxnet model, which penalizes large coefficients in computing maximum likelihoods. This method of feature selection reduces the set of covariates by possibly assigning some coefficients as zero, effectively negating them. In our case, zero coefficients were assigned to 53 of 61 genes. By comparing a basic predictor (1NN), we have shown that overall predictive power exists with both the 61 and 8 gene set. The advantage of feature selection is most apparent when binarizing inputs of gene selection for survival analysis. Analyzing the entire 61-gene set reveals only minor differences in median survival. However, survival differences are magnified when considering a restricted set of genes (see Figs 1 and 3). Thus we retain the overall accuracy of the analysis after reducing the number of genes, but improve upon the survival-predicting power of the procedure.

### Iron Genes as Predictors in GBM (WHO Grade IV)

The effects observed in LGG were not seen in glioblastoma using the available datasets. None of the three gene sets tested revealed a statistically significant difference in survival when considering a one-sided null hypothesis that aberrations cause decreased survival (although, surprisingly, aberrations in eight gene are associated with *increased* survival when considering a two sided test). However, the Coxnet feature selection algorithm returned a set of genes which are all contained in the reduced set of LGG genes. The failure of this panel to predict survival limits its utility, but the unsupervised selection of these genes in an independent dataset suggests a biological role for these genes in multiple cancers. The fact that almost all genes common to both glioma sets are also present in the 16-gene IRGS developed in breast cancer hints at a process that may be common to multiple neoplasms.

It is not clear why iron-regulating gene function fails to differentiate long- and short-term survivors in glioblastoma multiforme, especially considering the evidence that polymorphisms in a single iron-regulatory gene, *HFE*, may decrease survival in GBM [16]. Furthermore, it should be noted that TFRC has also been shown to negatively predict survival in GBM in other studies [36]. Clearly, some aspects of aberrant iron metabolism are relevant to the aggression of GBM, but the methods employed here were not able to identify a useful panel using the 61 iron regulatory genes in our starting set. The presence of both *IDH1*-mutated and *IDH1*-wild type individuals introduces considerable variability into the dataset, since these two tumor types appear to represent divergent biological origins [42,43]. Iron regulatory genes may provide useful prognostic and predictive information in one subtype, but given the relatively small proportions of patients with *IDH1* mutations, the present dataset did not provide a sufficient sample size to ascertain statistically meaningful conclusions. In addition, since TFRC and ferritin changes appear to be associated with cancer stem cell populations, it may be more appropriate to apply these methods to gene sets designed to interrogate stem cell character rather than iron aberrance alone in GBM.

The eight gene model generated by the Coxnet analysis provides a predictive tool with few enough elements to be potentially useful in future clinical applications. In conjunction with existing methods for predicting tumor behavior, it may provide additional prognostic and predictive insight. In addition, its underpinnings in iron biology may serve to focus research efforts in that field on these genes, which appear to have the most significant effects on survival for LGGs.

## Conclusions

We have showed that an eight gene signature derived from iron handling genes correlates with survival in LGG. A Coxnet model successfully restricted genes within the IRGS, and Kaplan-Meier survival curve analysis showed that these genes performed substantially better than the original set. Moreover, reducing the number of genes to eight preserves the underlying predictive power of the IRGS as shown by 1NN and random selection models. This work, on data from LGG patients, supports recent research showing iron metabolism affects tumor progression in multiple types of cancers. Furthermore, the genes identified in this study are risk factors in other cancers, suggesting that targeting these genes could provide therapeutic and prognostic tools with utility beyond LGG. The present study has showed that generating an unsupervised model for predicting survival using a biologically-defined group of genes can provide insight of both clinical and scientific interest.

## Supporting Information

S1 Text Description of IRGS genes. (DOCX)
