# Supplementary material for: Aberrations in the Iron Regulatory Gene Signature Are Associated with Decreased Survival in Diffuse Infiltrating Gliomas
Source: PLoS One. 2016 Nov 29;11(11):e0166593. doi: 10.1371/journal.pone.0166593 (PMC5127508; doi:10.1371/journal.pone.0166593)
Supplement: S1 Text — (DOCX) [file pone.0166593.s001.docx]

#

# Supporting Information: Description of IRGS

# Gene

### Other names

### Mutation

### Location

### Relations

### Function

### Expression in embryonic tissues and stem cells

### Associated diseases

### Entrez

### Papers

#

# CYBRD1: Cytochrome B Reductase 1

### Other names

DCYTB, FRRS3, CYB561A2

### Mutation

mRNA upregulation in 2% LGG cbioportal

### Location

Chromosome 2, NC_000002.12. Member of the cytochrome b(561) family that encodes an iron-regulated protein. It highly expressed in the duodenal brush border membrane. It has ferric reductase activity and is believed to play a physiological role in dietary iron absorption.

### Relations

GO annotations related to this gene include oxidoreductase activity, oxidizing metal ions and ferric-chelate reductase activity. An important paralog of this gene is CYB561

### Function

Ferric-chelate reductase that reduces Fe(3+) to Fe(2+). Present at the brush border of duodenal enterocytes where it probably reduces dietary Fe(3+) thereby facilitating its transport into the mucosal cells. Uses ascorbate as electron donor. May be involved in extracellular ascorbate recycling in erythrocyte membranes. May also act as a ferrireductase in airway epithelial cells.

### Expression in embryonic tissues and stem cells

In the Brain, it is expressed in: Metencephalon, Primitive Spinal Cord, and Hypothalamus

### Associated diseases

Hemochromatosis, iron deficiency, iron overload

### Entrez

<http://www.ncbi.nlm.nih.gov/gene?cmd=Retrieve&dopt=full_report&list_uids=79901>

### Papers

“When applied to colorectalcancer, we identified a missense polymorphism in iron-absorption gene CYBRD1 that associated with disease in individuals of English, but not Scottish, ancestry.” <http://www.ncbi.nlm.nih.gov/pubmed/23236349>

“Perl's staining showed increased iron in colorectal cancers, and there was a corresponding overexpression of components of the intracellular iron import machinery (DCYTB, DMT1, and TfR1).” <http://www.ncbi.nlm.nih.gov/pubmed/16641131>

Mechanistic analysis of iron accumulation by endothelial cells of the BBB <http://www.ncbi.nlm.nih.gov/pubmed/22434419>

# STEAP1: Six Transmembrane Epithelial Antigen Of The Prostate

### Other names

STEAP, PRSS24

### Mutation

mRNA Upregulation in 9% LGG cbioportal

### Location

Chromosome 7, NC_000007.14. This gene is predominantly expressed in prostate tissue, and is found to be upregulated in multiple cancer cell lines. The gene product is predicted to be a six-transmembrane protein, and was shown to be a cell surface antigen significantly expressed at cell-cell junctions.

### Relations

GO annotations related to this gene includetransporter activity and channel activity. An important paralog of this gene is STEAP2.

### Function

Metalloreductase that has the ability to reduce both Fe(3+) to Fe(2+) and Cu(2+) to Cu(1+). Uses NAD(+) as acceptor (By similarity).

### Expression in embryonic tissues and stem cells

Expressed in the brain in: Cranial Neural Crest Cells Branchial Arch 1, PureStem MEL2, NCr-fac Progenitor, Mesencephalic Floor Plate, Mature Choroid Plexus Cells

### Associated diseases

Prostate cancer

### Entrez

<http://www.ncbi.nlm.nih.gov/gene?cmd=Retrieve&dopt=full_report&list_uids=26872>

### Papers

“We found that CBX7 negatively or positively regulates the expression of several genes (such as SPP1, SPINK1, STEAP1, and FOS, FOSB, EGR1, respectively) associated to cancer progression, by interacting with their promoter regions and modulating their transcriptional activity. “<http://www.ncbi.nlm.nih.gov/pubmed/24865347>

“STEAP1 overexpression was detected in PCa, and was significantly associated with high-grade Gleason scores, seminal vesicle invasion, BCR, and worse outcome (metastasis or PCa-specific death). STEAP1 overexpression was significantly associated with shorter BCR-free survival. Multivariate analysis revealed that STEAP1 is an independent marker for BCR.” <http://www.ncbi.nlm.nih.gov/pubmed/24025158>

STEAP1 is overexpressed in cancers: a promising therapeutic target. <http://www.ncbi.nlm.nih.gov/pubmed/23142226>

STEAP proteins: from structure to applications in cancer therapy. <http://www.ncbi.nlm.nih.gov/pubmed/22522456>

# STEAP2: Six Transmembrane Epithelial Antigen Of The Prostate 2

### Other names

STMP, IPCA1, PUMPCn; STAMP1; PCANAP1

### Mutation

mRNA upregulation in 4% of LGG cbioportal

### Location

Chromosome 7, NC_000007.14. This gene is a member of the STEAP family and encodes a multi-pass membrane protein that localizes to the Golgi complex, the plasma membrane, and the vesicular tubular structures in the cytosol. A highly similar protein in mouse has both ferrireductase and cupric reductase activity, and stimulates the cellular uptake of both iron and copper in vitro. Increased transcriptional expression of the human gene is associated with prostate cancer progression. Alternate transcriptional splice variants, encoding different isoforms, have been characterized.

### Relations

GO annotations related to this gene include oxidoreductase activity and transporter activity. An important paralog of this gene is STEAP1B.

### Function

Metalloreductase that has the ability to reduce both Fe(3+) to Fe(2+) and Cu(2+) to Cu(1+). Uses NAD(+) as acceptor (By similarity).

### Expression in embryonic tissues and stem cells

Adult Oligodendrocyte Precursor Cells Lateral Ventricle

### Associated diseases

Prostate cancer

### Entrez

<http://www.ncbi.nlm.nih.gov/gene?cmd=Retrieve&dopt=full_report&list_uids=261729>

### Papers

Whole Transcriptome Sequencing Reveals Extensive Unspliced mRNA in Metastatic Castration-Resistant Prostate Cancer. <http://www.ncbi.nlm.nih.gov/pubmed/25189356>

“In relation to the STEAP family of metalloreductases, Steap2 is highly expressed in the brain and co-localizes with TfR; these observations make Steap2 a likely hBMVEC ferrireductase candidate.” <http://www.ncbi.nlm.nih.gov/pmc/articles/PMC3384751/>

The Steap proteins are metalloreductases. <http://www.ncbi.nlm.nih.gov/pubmed/16609065>

# HFE: Hereditary Hemochromatosis Gene

### Other names

HH, HFE1, HLA-H, MVCD7, TFQTL2

### Mutation

mRNA upregulation in 3% of LGG

### Location

Chromosome 8, NC_000008.11. The protein encoded by this gene is a membrane protein that is similar to MHC class I-type proteins and associates with beta2-microglobulin (beta2M). It is thought that this protein functions to regulate iron absorption by regulating the interaction of the transferrin receptor with transferrin. The iron storage disorder, hereditary haemochromatosis, is a recessive genetic disorder that results from defects in this gene. At least nine alternatively spliced variants have been described for this gene. Additional variants have been found but their full-length nature has not been determined.

### Relations

GO annotations related to this gene include peptide antigen binding and receptor binding. An important paralog of this gene is HLA-F.

### Function

Binds to transferrin receptor (TFR) through the extracellular domain in a pH-dependent manner and reduces its affinity for iron-loaded transferrin.

### Expression in embryonic tissues and stem cells

Not listed

### Associated diseases

hemochromatosis, microvascular complications of diabetes 7, hfe-associated hereditary hemochromatosis, porphyria cutanea tarda, hemosiderosis, nonalcoholic steatohepatitis, hfe hemochromatosis, modifier of cerebrooculofacioskeletal syndrome 2, variegate porphyria, hemochromatosis type 3, iron metabolism disease, alzheimer's disease, hemophilic arthropathy, siderosis, pyruvate kinase deficiency, lead poisoning, arthropathy, wilson disease, beta thalassemia, porphyria, thalassemia, liver disease, myelodysplastic syndromes, hepatocellular carcinoma

### Entrez

<http://www.ncbi.nlm.nih.gov/gene/3077>

### Papers

Expression of iron-related genes in human brain and brain tumors: <http://www.ncbi.nlm.nih.gov/pubmed/19386095>

HFE polymorphisms influence the response to chemotherapeutic agents via induction of p16INK4A: <http://www.ncbi.nlm.nih.gov/pubmed/21190189>

High frequency of the H63D mutation of the hemochromatosis gene (HFE) in malignant gliomas: <http://www.ncbi.nlm.nih.gov/pubmed/11591868>

C282Y-HFE gene variant affects cholesterol metabolism in human neuroblastoma cells: <http://www.ncbi.nlm.nih.gov/pubmed/24533143>

Inhibition of β2-microglobulin/hemochromatosis enhances radiation sensitivity by induction of iron overload in prostate cancer cells: <http://www.ncbi.nlm.nih.gov/pubmed/23874600>

The hemochromatosis proteins HFE, TfR2, and HJV form a membrane-associated protein complex for hepcidin regulation: http://www.ncbi.nlm.nih.gov/pubmed/22728873

# SCARA5: Scavenger Receptor Class A Member

### Other names

Tesr, NET33

### Mutation

Mutation occurs in 2% of LGG cbioportal cases, 1 amplification and the rest mRNA upregulation

### Location

Chromosome 8, NC_000008.11. SCARA5 (scavenger receptor class A, member 5 (putative)) is a protein-coding gene. Diseases associated with SCARA5 include placenta praevia, and placenta accreta.

### Relations

GO annotations related to this gene include ferritin receptor activity and scavenger receptor activity. An important paralog of this gene is MARCO.

### Function

Ferritin receptor that mediates non-transferrin-dependent delivery of iron. Mediates cellular uptake of ferritin-bound iron by stimulating ferritin endocytosis from the cell surface with consequent iron delivery within the cell. Delivery of iron to cells by ferritin is required for the development of specific cell types, suggesting the existence of cell type-specific mechanisms of iron traffic in organogenesis, which alternatively utilize transferrin or non-transferrin iron delivery pathways. Ferritin mediates iron uptake in capsule cells of the developing kidney. Binds preferrentially ferritin light chain (FTL) compared to heavy chain (FTH1) (By similarity).

### Expression in embryonic tissues and stem cells

Dermis, Heart, Adipose

### Associated diseases

Placenta previa and placenta accreta

### Entrez

<http://www.ncbi.nlm.nih.gov/gene/286133>

### Papers

Scara5 is a Ferritin Receptor Mediating Non-Transferrin Iron Delivery: <http://www.ncbi.nlm.nih.gov/pmc/articles/PMC2652503/>

“The most over-expressed gene in the UPS-A subset (the good prognosis group) was SCARA5, scavenger receptor class A, member 5, a gene that may act as a tumor suppressor in some models”: <http://www.ncbi.nlm.nih.gov/pmc/articles/PMC4082412/>

# LTF: Lactotransferrin

### Other names

### Mutation

Mutation occurs in 3% of LGG cbioportal cases, 3 homozygous deletion and the rest mRNA upregulation

### Location

Chromosome 3, NC_000003.12. This gene is a member of the transferrin family of genes and its protein product is found in the secondary granules of neutrophils. The protein is a major iron-binding protein in milk and body secretions with an antimicrobial activity, making it an important component of the non-specific immune system. The protein demonstrates a broad spectrum of properties, including regulation of iron homeostasis, host defense against a broad range of microbial infections, anti-inflammatory activity, regulation of cellular growth and differentiation and protection against cancer development and metastasis. Alternatively spliced transcript variants encoding different isoforms have been found for this gene.

### Relations

GO annotations related to this gene include ferric iron binding and serine-type endopeptidase activity. An important paralog of this gene is MFI2.

### Function

Transferrins are iron binding transport proteins which can bind two Fe(3+) ions in association with the binding of an anion, usually bicarbonate. Lactotransferrin is a major iron-binding and multifunctional protein found in exocrine fluids such as breast milk and mucosal secretions. Has antimicrobial activity, which depends on the extracellular cation concentration. Antimicrobial properties include bacteriostasis, which is related to its ability to sequester free iron and thus inhibit microbial growth, as well as direct bactericidal properties leading to the release of lipopolysaccharides from the bacterial outer membrane.

### Expression in embryonic tissues and stem cells

In the Brain: Myelinating Oligodendrocyte Cells, Forebrain White Matter

### Associated diseases

Mastitis, Ulcerative colitis, Diarrhea, Periodontal disease, Crohn's disease, Vasculitis, Keratoconjunctivitis sicca, Pancreatitis, Cancer, Perinatal necrotizing enterocolitis, Dental caries, Arthritis, Hypersensitivity reaction type II disease, Blepharitis, Candidiasis, Irritable bowel syndrome

### Entrez

<http://www.ncbi.nlm.nih.gov/gene/4057>

### Papers

“Lactotransferrin (LTF), a transcription factor which modulates cell growth and a variety of cellular and immune responses,[45](http://www.ncbi.nlm.nih.gov/pmc/articles/PMC2843477/#B45-8503) is down-regulated in malignant gliomas and nasopharyngeal carcinoma….We postulate here that Annexin A1, GPNMB, S100A11, LTF, RND3, and SFRP4 are candidate markers of subependymal giant cell astrocytomas. GPNMB, LTF, RND3, and SFRP4 may be responsible for benign phenotype and relatively low invasiveness of these tumors.” <http://www.ncbi.nlm.nih.gov/pmc/articles/PMC2843477/>

*In Vitro* and *in Vivo* Evaluation of Lactoferrin-Conjugated Liposomes as a Novel Carrier to Improve the Brain Delivery: <http://www.ncbi.nlm.nih.gov/pmc/articles/PMC3588019/>

# TFRC:

### Other names

T9, TR, TFR, p90, CD71, TFR1, TRFR

### Mutation

Mutation occurs in 5% of LGG cbioportal cases: 3 are amplified, 3 are homozygous deletions, 2 are missense mutations, 5 have upregulated RPPA only, 4 have upregulated mRNA and RPPA, and the rest have only upregulated mRNA.

### Location

Chromosome 3, NC_000003.12.

### Relations

GO annotations related to this gene include transferrin receptor activity and identical protein binding. An important paralog of this gene is NAALADL1.

### Function

Cellular uptake of iron occurs via receptor-mediated endocytosis of ligand-occupied transferrin receptor into specialized endosomes. Endosomal acidification leads to iron release. The apotransferrin-receptor complex is then recycled to the cell surface with a return to neutral pH and the concomitant loss of affinity of apotransferrin for its receptor. Transferrin receptor is necessary for development of erythrocytes and the nervous system (By similarity). A second ligand, the heditary hemochromatosis protein HFE, competes for binding with transferrin for an overlapping C-terminal binding site

### Expression in embryonic tissues and stem cells

Brain: Endothelial Cells, Mature Endothelial cells, and Adult Endothelial Cells in b lood-brain barrier; Mature brain microvascular endothelial cells

### Associated diseases

Anemia, Hemochromatosis, Nutrition disease, Thalassemia, Leukemia, Lymphoma

### Entrez

<http://www.ncbi.nlm.nih.gov/gene/7037>

### Papers

“Generally, the lowest expression levels with the lowest variability of candidate genes were seen for normal brain when compared with tumor tissue; this difference was statistically significant in 6 candidate genes: RPLP0, ACTB, PBGD, B2M, TFRC, and GUSB.” <http://www.ncbi.nlm.nih.gov/pmc/articles/PMC2940642/>

“TFRC, transferrin receptor, is known to be expressed in many tumor types (Table S3). Expression of VEGFA andTFRC is commonly regulated by HIF and MYC, which promote angiogenesis and proliferation, respectively.” <http://www.ncbi.nlm.nih.gov/pmc/articles/PMC3382591/#!po=62.5000>

Imaging Tumor Burden in the Brain withv89Zr-Transferrin: <http://www.ncbi.nlm.nih.gov/pmc/articles/PMC3747823/>

“The present study describes the expression of several iron-related genes including hepcidin (HAMP), HFE, neogenin(NEO1), transferrin receptor 1 (TFRC), transferrin receptor 2 (TFR2), and hemojuvelin(HFE2) in normal human brain, brain tumors, and astrocytoma cell lines. Our results suggested that all these genes except forHFE2 are expressed in the normal brain, and that their expression may be dysregulated in certain brain tumors.” <http://www.ncbi.nlm.nih.gov/pmc/articles/PMC2679039/>

Neural and oligodendrocyte progenitor cells: transferrin effects on cell proliferation. <http://www.ncbi.nlm.nih.gov/pmc/articles/PMC3592559/>

Identification of prognostic gene signatures of glioblastoma: a study based on TCGA data analysis: <http://www.ncbi.nlm.nih.gov/pmc/articles/PMC3688008/>

# SLC40A1: Solute Carrier Family 40 (Iron-Regulated Transporter), Member 1 AKA Ferroportin Gene

### Other names

FPN1, HFE4, MTP1, IREG1, MST079, MSTP079, SLC11A3

### Mutation

Mutation occurs in 3% of LGG cbioportal cases: all are upregulated mRNA.

### Location

Chromosome 2, NC_000002.12. The protein encoded by this gene is a cell membrane protein that may be involved in iron export from duodenal epithelial cells. Defects in this gene are a cause of hemochromatosis type 4 (HFE4).

### Relations

GO annotations related to this gene include iron ion transmembrane transporter activity.

### Function

May be involved in iron export from duodenal epithelial cell and also in transfer of iron between maternal and fetal circulation. Mediates iron efflux in the presence of a ferroxidase (hephaestin and/or ceruloplasmin).

### Expression in embryonic tissues and stem cells

Neural Crest: PureStem MEL2, NCr-fac Progenitor: Positive expression

Neural Crest: PureStem SM30, NCr-fac & Meso-latp Progenitor

### Associated diseases

Hemochromatosis, Anemia

### Entrez

<http://www.ncbi.nlm.nih.gov/gene/30061>

### Papers

Evidence for the multimeric structure of ferroportin: <http://www.ncbi.nlm.nih.gov/pmc/articles/PMC3688008/>

Glial Cell Ceruloplasmin and Hepcidin Differentially Regulate Iron Efflux from Brain Microvascular Endothelial Cells: <http://www.ncbi.nlm.nih.gov/pmc/articles/PMC3923066/>

Astrocytes derived from trisomic human embryonic stem cells express markers of astrocytic cancer cells and premalignant stem-like progenitors: <http://www.ncbi.nlm.nih.gov/pmc/articles/PMC2873256/>

# ISCU: Iron-Sulfur Cluster Assembly

### Other names

HML, ISU2, NIFU, NIFUN, hnifU, 2310020H20Rik

### Mutation

Mutation occurs in 3% of LGG cbioportal cases: 2 are amplified, 3 are homozygous deletions, and the rest are upregulated mRNA.

### Location

Chromosome 12, NC_000012.12. Iron-sulfur (Fe-S) clusters are necessary for several mitochondrial enzymes and other subcellular compartment proteins. They contain sulfur and iron, and are created via several steps that include cysteine desulfurases, iron donors, chaperones, and scaffold proteins. This gene encodes the two isomeric forms, ISCU1 and ISCU2, of the Fe-S cluster scaffold protein. Mutations in this gene have been found in patients with myopathy with severe exercise intolerance and myoglobinuria.

### Relations

GO annotations related to this gene include iron-sulfur cluster bindingand iron ion binding.

### Function

Involved in the assembly or repair of the [Fe-S] clusters present in iron-sulfur proteins. Binds iron.

### Expression in embryonic tissues and stem cells

Brain: Cerebral Cortex

### Associated diseases

Friedreich ataxia, Sideroblastic anemia, Lactic acidosis

### Entrez

<http://www.ncbi.nlm.nih.gov/gene/23479>

### Papers

“In some cells HIF-signaling is also known to regulate cellular metabolism by up-regulating the expression of the glycolytic genes and down-regulating mitochondrial activity by transactivating PDK1, a repressor of Pyruvate dehydrogenase and up-regulating miR-210, a suppressor of the iron-sulfur cluster assembly protein (ISCU).” <http://www.ncbi.nlm.nih.gov/pmc/articles/PMC3129496/>

“In addition, miR-210 most likely decreases the expression of ISCU (iron-sulfur cluster scaffold homolog) and COX10 (cytochrome c oxidase assembly protein), two key factors of the mitochondria electron transport chain and the tricarboxylic acid cycle, during hypoxia.” <http://www.ncbi.nlm.nih.gov/pmc/articles/PMC3200203/>

# SFXN1: Sideroflexin 1 AKA Tricarboxylate Carrier Protein

### Other names

f; 2810002O05Rik; A930015P12Rik

### Mutation

Mutation occurs in 7% of LGG cbioportal cases: 1 is amplified, 1 is a homozygous deletion, 11 are upregulated mRNA and the rest are downregulated mRNA.

### Location

Chromosome 13, NC_000079.6. SFXN1 (sideroflexin 1) is a protein-coding gene.

### Relations

GO annotations related to this gene include cation transmembrane transporter activity. An important paralog of this gene is SFXN4.

### Function

Might be involved in the transport of a component required for iron utilization into or out of the

mitochondria.

### Expression in embryonic tissues and stem cells

Neurons: Mature Horizonal Cells - Inner Nuclear Layer

### Associated diseases

Aceruloplasminemia

### Entrez

<http://www.ncbi.nlm.nih.gov/gene/14057>

### Papers

“The differential expression across age of the two mitochondrial genes Sfxn1 and Tomm20 in Glud1 vs. wt mouse hippocampi might be further evidence of functional differences between wt and Tg mouse brain mitochondria.” <http://www.ncbi.nlm.nih.gov/pmc/articles/PMC3973933/>

# EPAS1: Endothelial PAS Domain Protein

### Other names

HLF; MOP2; ECYT4; HIF2A; PASD2; bHLHe73

### Mutation

Mutation occurs in 5% of LGG cbioportal cases: 1 is missense mutated and the rest are upregulated mRNA.

### Location

Chromosome 2, NC_000002.12. This gene encodes a transcription factor involved in the induction of genes regulated by oxygen, which is induced as oxygen levels fall. The encoded protein contains a basic-helix-loop-helix domain protein dimerization domain as well as a domain found in proteins in signal transduction pathways which respond to oxygen levels. Mutations in this gene are associated with erythrocytosis familial type 4.

### Relations

GO annotations related to this gene include sequence-specific DNA bindingand transcription factor binding. An important paralog of this gene is ARNT2.

### Function

Transcription factor involved in the induction of oxygen regulated genes. Binds to core DNA sequence 5'-[AG]CGTG-3' within the hypoxia response element (HRE) of target gene promoters. Regulates the vascular endothelial growth factor (VEGF) expression and seems to be implicated in the development of blood vessels and the tubular system of lung. May also play a role in the formation of the endothelium that gives rise to the blood brain barrier. Potent activator of the Tie-2 tyrosine kinase expression. Activation seems to require recruitment of transcriptional coactivators such as CREBPB and probably EP300. Interaction with redox regulatory protein APEX seems to activate CTAD.

### Expression in embryonic tissues and stem cells

Inner Mass Cells, Liver, Placenta, Umbilical Endothelium, Heart

### Associated diseases

Multiple paragangliomas associated with polycythemia, sporadic pheochromocytoma, sporadic secreting paraganglioma, familial erythrocytosis 4, vein disease, nodular malignant melanoma, erythrocytosis, hypoxia, somatostatinoma, pheochromocytoma, renal cell carcinoma

### Entrez

<http://www.ncbi.nlm.nih.gov/gene/2034>

### Papers

A Macrophage Dominant PI-3K Isoform Controls Hypoxia Induced HIF1alpha & HIF2alpha Stability and Tumor Growth, Angiogenesis and Metastasis. <http://mcr.aacrjournals.org/content/early/2014/08/07/1541-7786.MCR-13-0682.long>

This large genetic analysis suggests that variants in VEGF-A, EPAS1, IL8RA, VHL, and VEGF-C have potential value in predicting bevacizumab treatment outcome across tumor types. <http://www.ncbi.nlm.nih.gov/pubmed/25012543>

Molecular analysis revealed that CBS knockdown subclones expressed higher basal levels of the transcriptional activator hypoxia-inducible factor 2alpha (HIF-2alpha/EPAS1). HIF-2alpha knockdown counteracted the effect of CBS knockdown on anchorage-independent growth. Bioinformatic analysis of mRNA expression data from human glioma specimens revealed a significant association between low expression of CBS mRNA and high expression of angiopoietin-like 4 (ANGPTL4) and vascular endothelial growth factor (VEGF) transcripts, which are HIF-2 target gene products that were also increased in CBS knockdown subclones. These results suggest that decreased CBS expression in glioma increases HIF-2alpha protein levels and HIF-2 target gene expression, which promotes glioma tumor formation. <http://www.ncbi.nlm.nih.gov/pubmed/24994751>

Hypoxia-inducible factor (HIF) is a heterodimeric complex, composed of oxygen-induced HIFα and constitutively expressed HIFβ subunits, which mediates the primary transcriptional response to hypoxic stress. Among HIFα isoforms, HIF1α (HIF1A) and endothelial PAS domain-containing protein 1 (EPAS1) are able to robustly activate hypoxia-responsive gene signatures. <http://www.ncbi.nlm.nih.gov/pubmed/24825851>

Oncostatin M-induced genes in human astrocytomas. <http://www.ncbi.nlm.nih.gov/pubmed/17982672>

# SLC25A37: Solute Carrier Family 25 AKA Mitochondrial Iron Transporter

### Other names

MSC; MFRN; MSCP; HT015; MFRN1; PRO1278; PRO1584; PRO2217

### Mutation

Mutation occurs in 4% of LGG cbioportal cases: all are upregulated mRNA.

### Location

Chromosome 8, NC_000008.11. SLC25A37 is a solute carrier localized in the mitochondrial inner membrane. It functions as an essential iron importer for the synthesis of mitochondrial heme and iron-sulfur clusters.

### Relations

GO annotations related to this gene include iron ion transmembrane transporter activity. An important paralog of this gene is SLC25A28.

### Function

Mitochondrial iron transporter that specifically mediates iron uptake in developing erythroid cells, thereby playing an essential role in heme biosynthesis. The iron delivered into the mitochondria, presumably as Fe(2+), is then probably delivered to ferrochelatase to catalyze Fe(2+) incorporation into protoprophyrin IX to make heme.

### Expression in embryonic tissues and stem cells

Bone, blood, and liver

### Associated diseases

Intrahepatic cholestasis, erythropoietic protoporphyria

### Entrez

<http://www.ncbi.nlm.nih.gov/gene/51312>

### Papers

Fourteen novel human members of mitochondrial solute carrier family 25 (SLC25) widely expressed in the central nervous system. <http://www.sciencedirect.com/science/article/pii/S0888754306001996>

ABCG2: ATP-binding cassette, sub-family G (WHITE), member 2

### Other names

MRX; MXR; ABCP; BCRP; BMDP; MXR1; ABC15; BCRP1; CD338; GOUT1; CDw338; UAQTL1; EST157481

### Mutation

Mutation occurs in 4% of LGG cbioportal cases: all are upregulated mRNA.

### Location

Chromosome 4, NC_000004.12. The membrane-associated protein encoded by this gene is included in the superfamily of ATP-binding cassette (ABC) transporters. ABC proteins transport various molecules across extra- and intra-cellular membranes. ABC genes are divided into seven distinct subfamilies (ABC1, MDR/TAP, MRP, ALD, OABP, GCN20, White). This protein is a member of the White subfamily. Alternatively referred to as a breast cancer resistance protein, this protein functions as a xenobiotic transporter which may play a major role in multi-drug resistance. It likely serves as a cellular defense mechanism in response to mitoxantrone and anthracycline exposure. Significant expression of this protein has been observed in the placenta, which may suggest a potential role for this molecule in placenta tissue. Multiple transcript variants encoding different isoforms have been found for this gene.

### Relations

GO annotations related to this gene include ATPase activity, coupled to transmembrane movement of substances and protein homodimerization activity. An important paralog of this gene is ABCG1.

### Function

High-capacity urate exporter functioning in both renal and extrarenal urate excretion. Plays a role in porphyrin homeostasis as it is able to mediates the export of protoporhyrin IX (PPIX) both from mitochondria to cytosol and from cytosol to extracellular space, and cellular export of hemin, and heme. Xenobiotic transporter that may play an important role in the exclusion of xenobiotics from the brain. Appears to play a major role in the multidrug resistance phenotype of several cancer cell lines. Implicated in the efflux of numerous drugs and xenobiotics: mitoxantrone, the photosensitizer pheophorbide, camptothecin, methotrexate, azidothymidine (AZT), and the anthracyclines daunorubicin and doxorubicin.

### Expression in embryonic tissues and stem cells

Adult Endothelial Cells Blood Brain Barrier

Mature Choroid Plexus Cells Choroid Plexus

Choroid Plexus Progenitor Cells Choroid Plexus

Mature Endothelial Cells Blood Brain Barrier

Cerebral Cortex

Mature brain microvascular endothelial cells

Nervous System Schwann Precursor Cells

Neural Tube Floor plate-like cells

### Associated diseases

Gout, Leukemia, Breast cancer, Hyperuricemia, Lung cancer, nonpapillary renal cell carcinoma, erythroplakia, choriocarcinoma, adult acute lymphocytic leukemia, dysembryoplastic neuroepithelial tumor, acute lymphocytic leukemia, acute myeloid leukemia

### Entrez

<http://www.ncbi.nlm.nih.gov/gene/9429>

### Papers

EGCG inhibits properties of glioma stem-like cells and synergizes with temozolomide through downregulation of P-glycoprotein inhibition. <http://www.ncbi.nlm.nih.gov/pubmed/25173233>

Downregulation of ABCG2 protein inhibits migration and invasion in U251 glioma stem cells. <http://www.ncbi.nlm.nih.gov/pubmed/24781949>

“ABCG2 is an ATP-binding cassette transporter protein, which was identified to be overexpressed in GSCs and higher-grade glioma tissues.” <http://www.ncbi.nlm.nih.gov/pubmed/24777293>

Melatonin-induced methylation of the ABCG2/BCRP promoter as a novel mechanism to overcome multidrug resistance in brain tumour stem cells. <http://www.ncbi.nlm.nih.gov/pubmed/23632480>

Dual mTORC1 and mTORC2 inhibitor Palomid 529 penetrates the blood-brain barrier without restriction by ABCB1 and ABCG2. <http://www.ncbi.nlm.nih.gov/pubmed/23436212>

Breast cancer resistance protein (BCRP/ABCG2) localises to the nucleus in glioblastoma multiforme cells. <http://www.ncbi.nlm.nih.gov/pubmed/22401348>

The ABCG2 transporter is a key molecular determinant of the efficacy of sonodynamic therapy with Photofrin in glioma stem-like cells. <http://www.ncbi.nlm.nih.gov/pubmed/22771084>

Establishment of prognostic models for astrocytic and oligodendroglial brain tumors with standardized quantification of marker gene expression and clinical variables.<http://www.ncbi.nlm.nih.gov/pubmed/21234290>

Glioma stem/progenitor cells contribute to neovascularization via transdifferentiation. <http://www.ncbi.nlm.nih.gov/pubmed/20697979>

The ABCG2 resistance network of glioblastoma. <http://www.ncbi.nlm.nih.gov/pubmed/19713741>

ABCG2 is related with the grade of glioma and resistance to mitoxantone, a chemotherapeutic drug for glioma. <http://www.ncbi.nlm.nih.gov/pubmed/19340456>

ABC transporters, cytochromes P450 and their main transcription factors: expression at the human blood-brain barrier. <http://www.ncbi.nlm.nih.gov/pubmed/19094056>

A General Map of Iron Metabolism and Tissue-specific Subnetworks <http://www.ncbi.nlm.nih.gov/pmc/articles/PMC2680238/>

# SFXN5:

### Other names

BBG-TCC

### Mutation

Mutation occurs in 3% of LGG cbioportal cases: 1 case is a missense mutation and all others are upregulated mRNA.

### Location

Chromosome 2, NC_000002.12. SFXN5 (sideroflexin 5) is a protein-coding gene.

### Relations

GO annotations related to this gene include cation transmembrane transporter activity andcitrate transmembrane transporter activity. An important paralog of this gene is SFXN4.

### Function

Transports citrate. Potential iron transporter.

### Expression in embryonic tissues and stem cells

Brain: Cerebral Cortex

### Associated diseases

None Identified

### Entrez

<http://www.ncbi.nlm.nih.gov/gene/94097>

### Papers

There were no relevant papers on SFXN5.

HIF1AN: Hypoxia Inducible Factor 1, Alpha Subunit Inhibitor

### Other names

FIH1

### Mutation

Mutation occurs in 13% of LGG cbioportal cases: 1 case is a homozygous deletion, 5 are upregulated mRNA, and the rest are downregulated mRNA.

### Location

Chromosome 10, NC_000010.11. HIF1AN (hypoxia inducible factor 1, alpha subunit inhibitor) is a protein-coding gene.

### Relations

GO annotations related to this gene include protein homodimerization activity and NF-kappaB binding. An important paralog of this gene is TYW5.

### Function

Hydroxylates HIF-1 alpha at 'Asp-803' in the C-terminal transactivation domain (CAD). Functions as an oxygen sensor and, under normoxic conditions, the hydroxylation prevents interaction of HIF-1 with transcriptional coactivators including Cbp/p300-interacting transactivator. Involved in transcriptional repression through interaction with HIF1A, VHL and histone deacetylases. Hydroxylates specific Asn residues within ankyrin repeat domains (ARD) of NFKB1, NFKBIA, NOTCH1, ASB4, PPP1R12A and several other ARD-containing proteins. Also hydroxylates Asp and His residues within ARDs of ANK1 and TNKS2, respectively. Negatively regulates NOTCH1 activity, accelerating myogenic differentiation. Positively regulates ASB4 activity, promoting vascular differentiation.

### Expression in embryonic tissues and stem cells

None listed

### Associated diseases

Hypoxia, pancreatic endocrine tumors, pre-eclampsia

### Entrez

<http://www.ncbi.nlm.nih.gov/gene/55662>

### Reactome

<http://www.reactome.org/PathwayBrowser/#DB=gk_current&FOCUS_SPECIES_ID=48887&FOCUS_PATHWAY_ID=1234174&ID=1234162>

### Papers

Inverse solvent isotope effects arising from substrate triggering in the factor inhibiting hypoxia inducible factor. <http://www.ncbi.nlm.nih.gov/pubmed/23351038>

“Overexpression of the hypoxia inducible factor 1 (HIF-1) and HIF-2 transcription factors and the consequent upregulation of hypoxia inducible mRNAs is a feature of many human cancers and may be unrelated to tissue hypoxia. Thus, the VHL (von Hippel-Lindau) tumour suppressor gene (TSG) regulates HIF-1 and HIF-2 expression in normoxia by targeting the alpha subunits for ubiquitination and proteolysis.” <http://www.ncbi.nlm.nih.gov/pubmed/15220362>

Hypoxia-inducible factor 1alpha/vascular endothelial growth factor axis in astrocytomas. Associations with microvessel morphometry, proliferation and prognosis. <http://www.ncbi.nlm.nih.gov/pubmed/?term=hif1an+glioma>

The Role of Factor Inhibiting HIF (FIH-1) in Inhibiting HIF-1 Transcriptional Activity in Glioblastoma Multiforme <http://www.ncbi.nlm.nih.gov/pmc/articles/PMC3900478/>

“In addition, we found that IDH1 mutated cells overexpress HIF1AN. The HIF1AN gene inhibits HIF1α. Since HIF1α acts as an oxygen sensor that promotes angiogenesis, the formation of dysfunctional tumor vasculature is counteracted inIDH1 mutated cells.” <http://www.ncbi.nlm.nih.gov/pmc/articles/PMC3896697/>

The asparaginyl hydroxylase FIH (Factor Inhibiting HIF) is an essential regulator of metabolism. <http://www.ncbi.nlm.nih.gov/pmc/articles/PMC2893150/>

HIF1α and HIF2α: sibling rivalry in hypoxic tumor growth and progression. <http://www.ncbi.nlm.nih.gov/pmc/articles/PMC3401912/>

FIH-1: a novel protein that interacts with HIF-1α and VHL to mediate repression of HIF-1 transcriptional activity: <http://www.ncbi.nlm.nih.gov/pmc/articles/PMC312814/>

Aspartyl-(asparaginyl) β-Hydroxylase, Hypoxia-Inducible Factor-1α and Notch Cross-Talk in Regulating Neuronal Motility <http://www.ncbi.nlm.nih.gov/pmc/articles/PMC3154035/>

ALAD: Aminolevulinate Dehydratase

### Other names

PBGS; ALADH

### Mutation

Mutation occurs in 4% of LGG cbioportal cases: all are upregulated mRNA.

### Location

Chromosome 9, NC_000009.12. The ALAD enzyme is composed of 8 identical subunits and catalyzes the condensation of 2 molecules of delta-aminolevulinate to form porphobilinogen (a precursor of heme, cytochromes and other hemoproteins). ALAD catalyzes the second step in the porphyrin and heme biosynthetic pathway; zinc is essential for enzymatic activity. ALAD enzymatic activity is inhibited by lead and a defect in the ALAD structural gene can cause increased sensitivity to lead poisoning and acute hepatic porphyria.

### Relations

GO annotations related to this gene include lead ion binding and identical protein binding.

### Function

Catalyzes an early step in the biosynthesis of tetrapyrroles. Binds two molecules of 5-aminolevulinate per subunit, each at a distinct site, and catalyzes their condensation to form porphobilinogen.

### Expression in embryonic tissues and stem cells

Cerebral Cortex, Cerebellum, Hippocampus, Lateral Ventricle

### Associated diseases

Acute hepatic porphyria, lead poisoning, chronic hepatic porphyria, hypochromic anemia, cutaneous porphyria, fanconi syndrome, tyrosinemia type i, hereditary coproporphyria, variegate porphyria, tyrosinemia, porphyria, porphyria cutanea tarda, uremia, acute porphyria, acute intermittent porphyria, erythropoietic protoporphyria, amyotrophic lateral sclerosis, malaria

### Entrez

<http://www.ncbi.nlm.nih.gov/gene/210>

### Papers

Comparison of occupational exposure assessment methods in a case-control study of lead, genetic susceptibility and risk of adult brain tumour <http://www.ncbi.nlm.nih.gov/pubmed/20798009>

Delta-aminolevulinic acid dehydratase polymorphism and risk of brain tumors in adults. <http://www.ncbi.nlm.nih.gov/pubmed/16140629>
